# Design and compatibility analysis of a solar panel integrated UHF antenna for nanosatellite space mission

**DOI:** 10.1371/journal.pone.0205587

**Published:** 2018-11-14

**Authors:** Touhidul Alam, Mohammad Tariqul Islam, Md. Amanath Ullah, Rahmi Rahmatillah, Kateryna Aheieva, Chow Chee Lap, Mengu Cho

**Affiliations:** 1 Centre of Advanced Electronic & Communication Engineering, Faculty of Engineering and Built Environment, Universiti Kebangsaan Malaysia, Bangi, Selangor D.E., Malaysia; 2 Laboratory of Spacecraft Environment Interaction Engineering (LaSEINE), Kyushu Institute of Technology, Kitakyushu-shi, Fukuoka, Japan; 3 School of Electrical Electronic Engineering, Nanyang Technological University, Nanyang Drive, Singapore; University of Scranton, UNITED STATES

## Abstract

A compact UHF antenna has been presented in this paper for nanosatellite space mission. A square ground plane with slotted rectangular radiating element have been used. Coaxial probe feeding is used to excite. The rectangular slot of the radiating patch is responsible for resonating at lower UHF bands. One of the square faces of the nanosatellite structure works as the ground plane for the slotted radiating element. The fabricated prototype of the proposed antenna has achieved an impedance bandwidth (S11< -10dB) of 7.0 MHz (398 MHz– 405 MHz) with small size of 97 mm× 90 mm radiating element. The overall ground plane size is 100 mm × 100 mm × 0.5 mm. The proposed antenna has achieved a gain of 1.18 dB with total efficiency of 62.5%. The proposed antenna addresses two design challenges of nanosatellite antenna, (a) assurance of the placement of solar panel beneath the radiating element; (b) providing about 50% open space for solar irradiance to pass onto the solar panel, enabling the solar panel to achieve up to 93.95% of power under of normal conditions.

## Introduction

With the advent of modern technology nanosatellites are flourishing a new dimension in space communication. The launch of first CubeSat in 2003 introduced a new era for scientific, private, and government missions, research interest of universities [1] and enabled researchers to visualize and plan bigger mission with less expense [2]. CubeSats missions are rapidly evolving as viable platform for Earth and space science. Simplicity, light weight, cost effectiveness and short development period make them ideal candidates for low earth orbit constellations. Nanosatellite Space missions are being fruitful in coastal and inland observation of flood, volcanic eruption, landslides, draught, forest fires, critical forecast of natural disaster, space atmosphere observation, monitoring agriculture and agri-environmental conditions. Nanosatellites are being designed in big scale for commercial, government and educational research because of continuous reduction of development cost, increased demand due to wide application range Moreover NASA space exploration missions have planned a telecom system for asteroid, lunar and planetary destinations by launching several CubeSats beyond earth orbit recently [3, 4].

To establish and maintain contact between the CubeSat and Earth, the communication system plays vital role and undoubtedly antennas are part and parcel of the communication system. Antenna design for nanosatellites specially for lower frequency presents its own challenge and has been a critical issue to the CubeSats researchers [5]. Besides, limited surface of the nanosatellite body is another major issue for solar panel integration [6]. So, to design an ultra-high frequency (UHF) antenna, strategically integrated with solar cells and without mechanical deployment requirements, has become a big challenge for nanosatellite and antenna research.

Size and weight of the CubeSat are two of the most crucial factors that have profound impact on antenna type and design. Researchers are focusing on several types of antennas that can be a comprise between the CubeSat requirement and performance of the antenna. Wire antennas are widely used on satellites. However, mechanical deployment of dipoles, monopoles, Yagi-Uda arrays and helical antennas is quite sophisticated and is liable to increase the chance of mission failure[7]. In [8], a monopole antenna has been presented for nanosatellite communication operating at 435–438 MHz. The length of the monopole antenna is 175 mm. Though the antenna achieved 2.35 dB gain it needs to be deployed externally which needs deployment circuitry that makes the whole system more sophisticated. Patch antennas are good replacement of wire antennas. Patch antenna provides low profile and improves the mission reliability in contrast to deployable antenna. However, patch antenna occupies nanosatellite body surface and make sufficient integration of solar cells a complex problem. To mitigate this problem transparent patch antennas have been addressed [9, 10] but it is difficult to obtain operating band in lower UHF due to design restriction. A printed patch antenna has been presented in [11] that operates at 427.38–437.17 MHz. The dimension the antenna is 320×80×3.17 mm3 which is very large in size in context of compatibility with 1U nanosatellite. In [12] a printed patch antenna design is illustrated operation at 410–485 MHz. This antenna is also too large in size with a dimension of 220×220×28.5 mm3. Horn antennas can serve with good radiation properties along with good gain. But, they are more suitable for higher frequency operation [13]. In addition, transparent antenna is one of the several approaches to mitigate the power scarcity in nanosatellites[14]. Solar panels can be placed below these types of antenna. Thus, it can serve the communication purpose along with allowing solar penetration at the same time. Up to 90% of transparency can be facilitated by transparent antennas. However, fabrication of transparent antenna can be complex and transparent substrate materials like Lexan or quartz are expensive.

A design of a 3D antenna for the application of UHF in CubeSats is proposed in this paper. Certain advantages of 3D antennas are considered here for the design proposal, namely, 3D antennas can use the whole volume independent of impedance matching, easier fabrication using 3D printing or stamping metal sheet method etc. The initial targeted bands are UHF 401MHz bands, which are commonly used for nanosatellites. As the frequency decreases, the antenna becomes larger in size. To address this issue, one face of the typical nanosatellite structure is considered as the ground plane for the main radiator. Moreover, the radiating element is designed to allow sufficient space for the placement of solar panel between the ground plane radiating element, that can facilitate with enough free space for solar irradiance penetration.

## Antenna design

**Fig 1:**
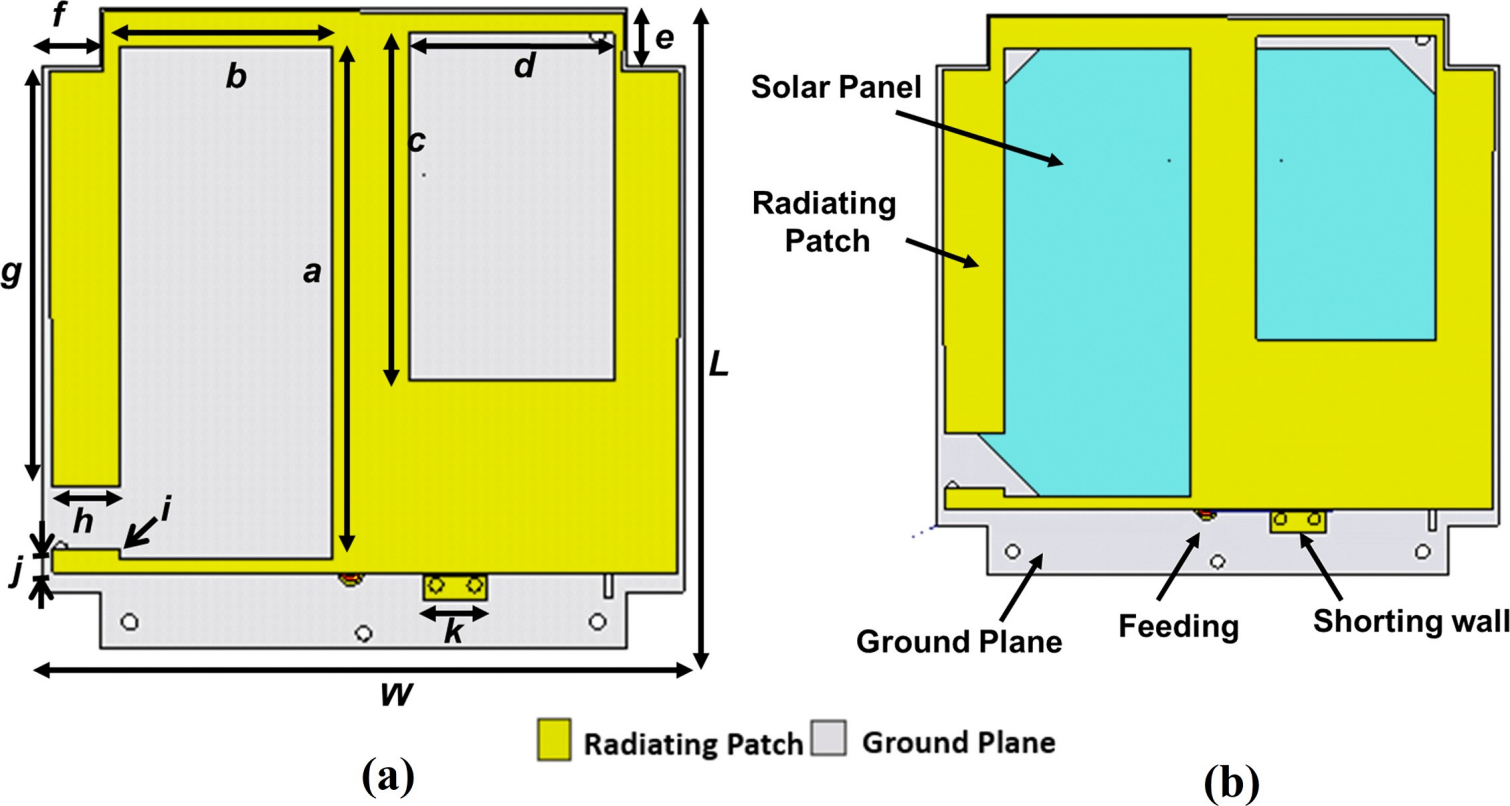
(a) Antenna Design Layout. (b). Antenna with Solar Panel.

The design layout of the proposed antenna is illustrated in Fig 1. The proposed antenna is designed considering the following issues-

- use satellite body as ground plane
- allow sufficient space for the placement of solar panels between the ground plane and radiating element of the proposed antenna.
- ensure more than fifty percent open space for solar irradiance penetration onto the face where antenna is attached.

The antenna is designed to avoid deployment complexity and to mitigate power scarcity considering the antenna design constraints of 1U, 2U nanosatellite structures. Initially, the following constraints were set to design the antenna.

1. The overall size of the proposed antenna is limited to 10×10×10 cm^3^.
2. The gap between ground plane and radiating structure should stay within 6 mm.

**Fig 2:**
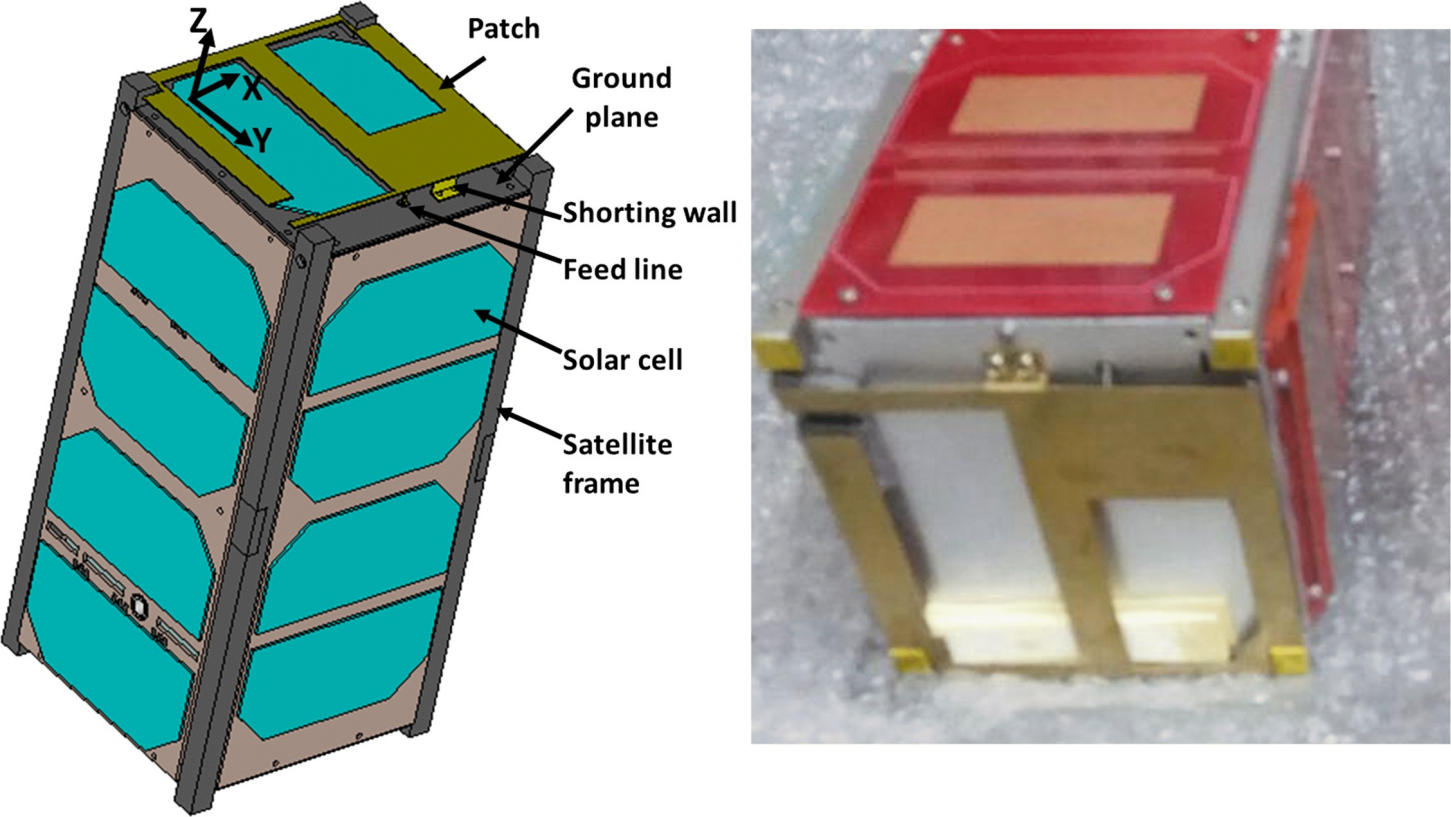
Antenna with 2U nanosatellite body.

The main radiating element is designed in a way that it can provide maximum effective length to achieve resonance at UHF 401MHz. The small radiating element at the top left side is mostly responsible for tuning resonance. The UHF antenna is designed using 0.5mm brass sheet for radiating element and 1.50 mm thick hard anodized Aluminum 6061 sheet is used as ground plane. The considered brass material in simulation has 2.74×10^7^ S/m electric conductivity, 109 W/K/m thermal conductivity and 0.38 kJ/K/kg heat capacity. The gap between ground plane and radiating structure optimized to 4.6 mm that provides sufficient space to the ground plane to be mounted on the CubeSat body and placement of solar panel, shown in Fig 2. The design parameters of the proposed antenna are enlisted in Table 1. The shorting wall technique is used to achieve resonance at lower frequency and better reflection coefficient [15, 16]. Gallium Arsenide based solar panels with dielectric constant of 12.94 have been utilized in simulation. The thermal conductivity and heat capacity of the panels are considered 54 W/K/m and 0.33 kJ/K/kg, respectively.

**Table 1:** Antenna parameters.

Parameters	Value (mm)
a	80
b	33
c	54
d	32
*e*	9.2
*f*	8.5
*g*	64.5
*h*	10.6
*i*	1.0
*j*	3.0
*k*	9.8
*L*	100
*W*	90

## Antenna performance analysis

**Fig 3:**
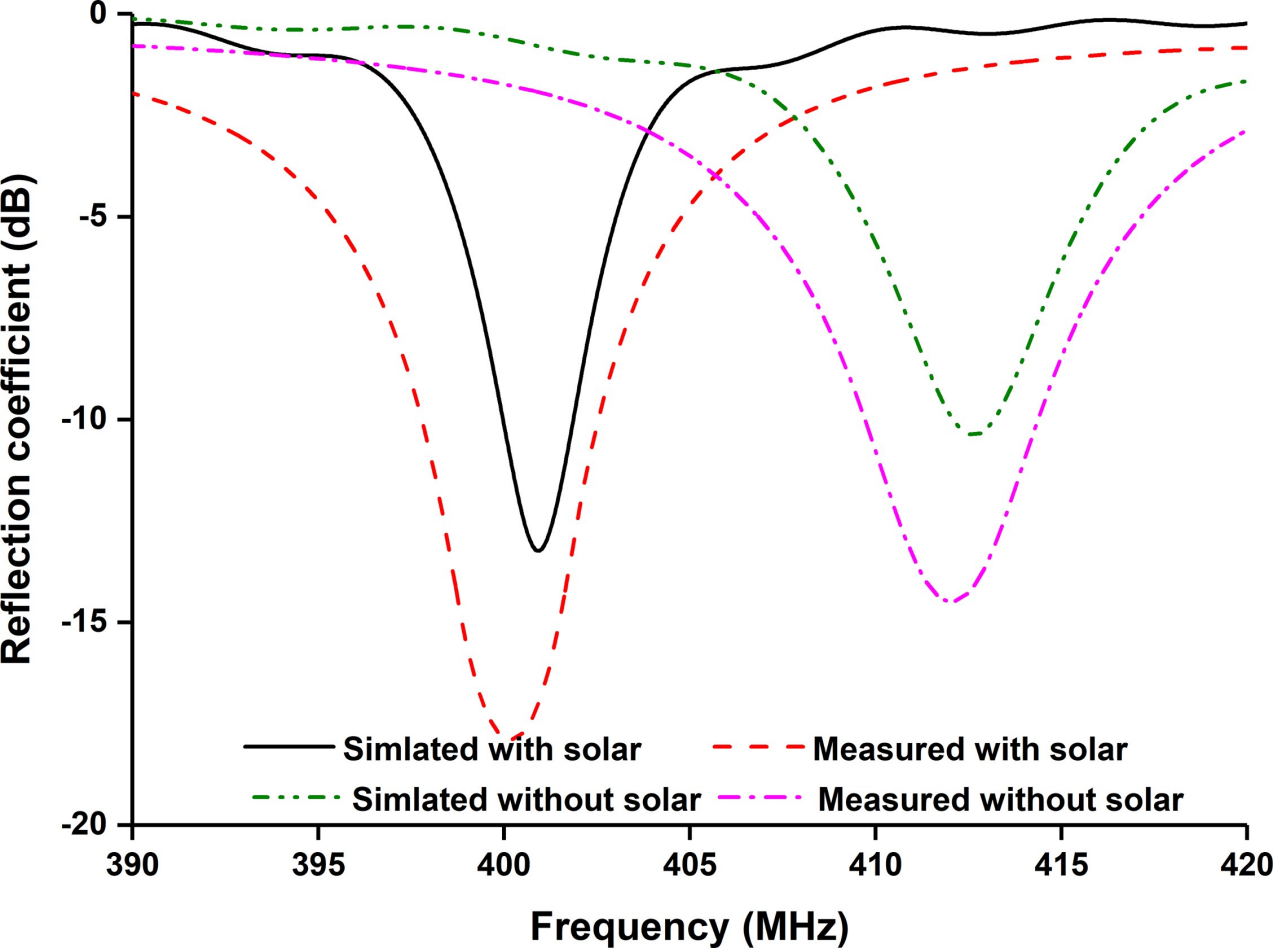
Simulated and measured reflection coefficient.

The proposed antenna has been fabricated according to the listed parameters in Table 1. The antenna attached to the nanosatellite structure, shown in Fig 2. It is shown from Fig 2 that the proposed antenna is compatible with both 1U and 2U nanosatellite structure. The reflection coefficient of the proposed antenna has been measured using performance network analyzer (PNA) Agilent N5227A. The has been calibrated before calibration to minimize the I/O port mismatch, connecting cable loss, etc. To do that, N4694A MW electronic calibration (ECal) module was used. The reflection coefficient has been analyzed with and without integrating solar panel, shown in Fig 3. Fig 3 shows that a good input match with magnitude of reflection coefficient ≤-15dB. The reflection coefficient bandwidth increases after solar cell placement due to the lossy properties of the solar cell. Moreover, it is noted that the solar cell affected the input impedance and the resonance shifts by 10 MHz to lower frequency [6]. This shifting effect in both simulation and measured results show good agreement.

**Fig 4:**
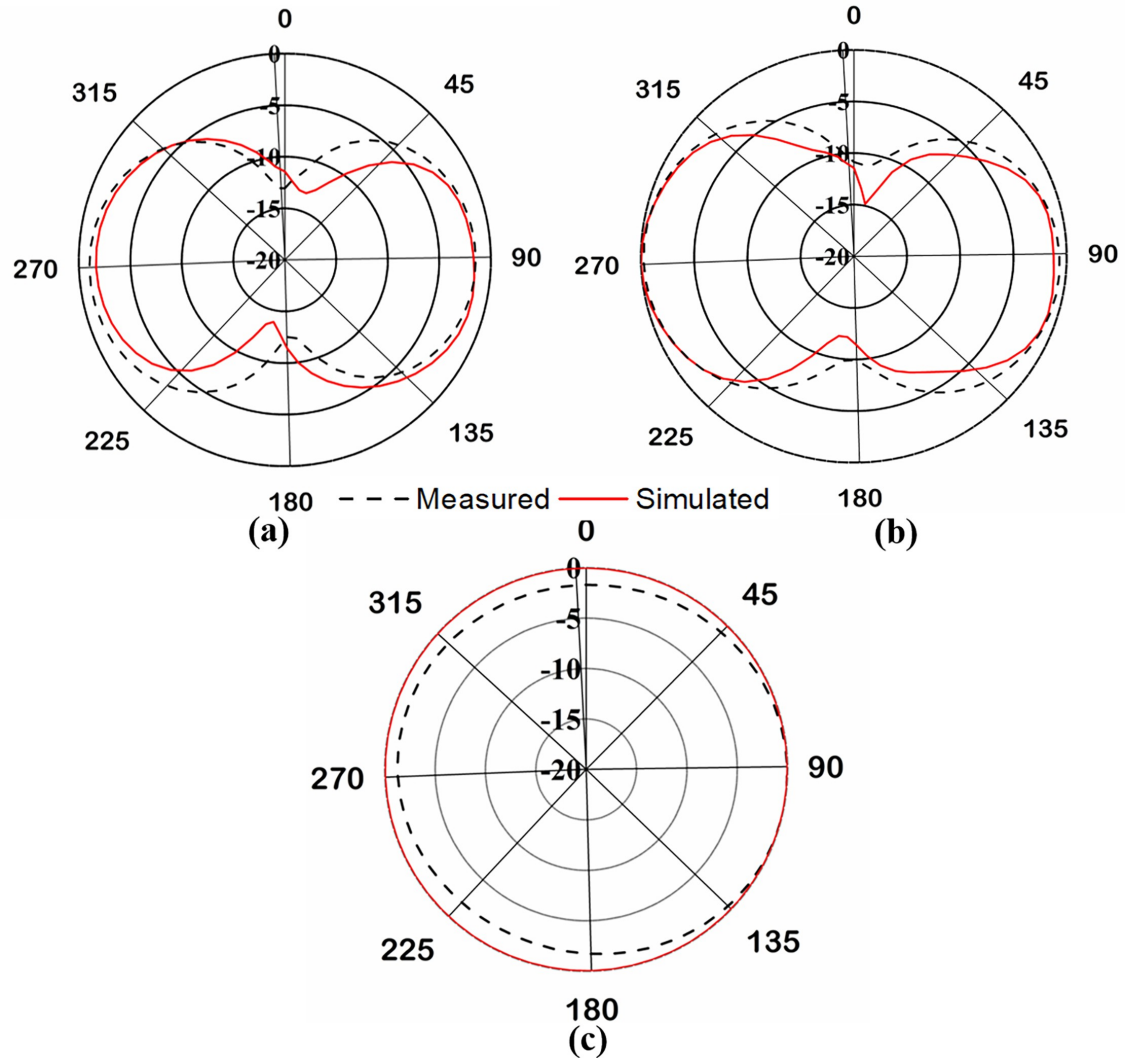
(a). Normalized radiation pattern at elevation plane 0˚. (b). Normalized radiation pattern at elevation plane 90˚. (c). Normalized radiation pattern at azimuth plane.

The radiation pattern of the proposed UHF antenna was measured in Satimo nearfield measurement systems. The radiation patterns are measured in both azimuth and elevation planes at 401MHz, illustrated in Fig 4. Fig 4(A) and Fig 4(B) depict the measured and simulated elevation radiation pattern at 0˚ and 90˚, respectively. Moreover, Fig 4(C) illustrates the simulated and measured azimuth radiation pattern, which shows Omni-directional radiation pattern. The radiation pattern is similar to the conventional planar inverted-F antenna. The both simulated and measured radiation pattern results are in good agreement.

**Fig 5:**
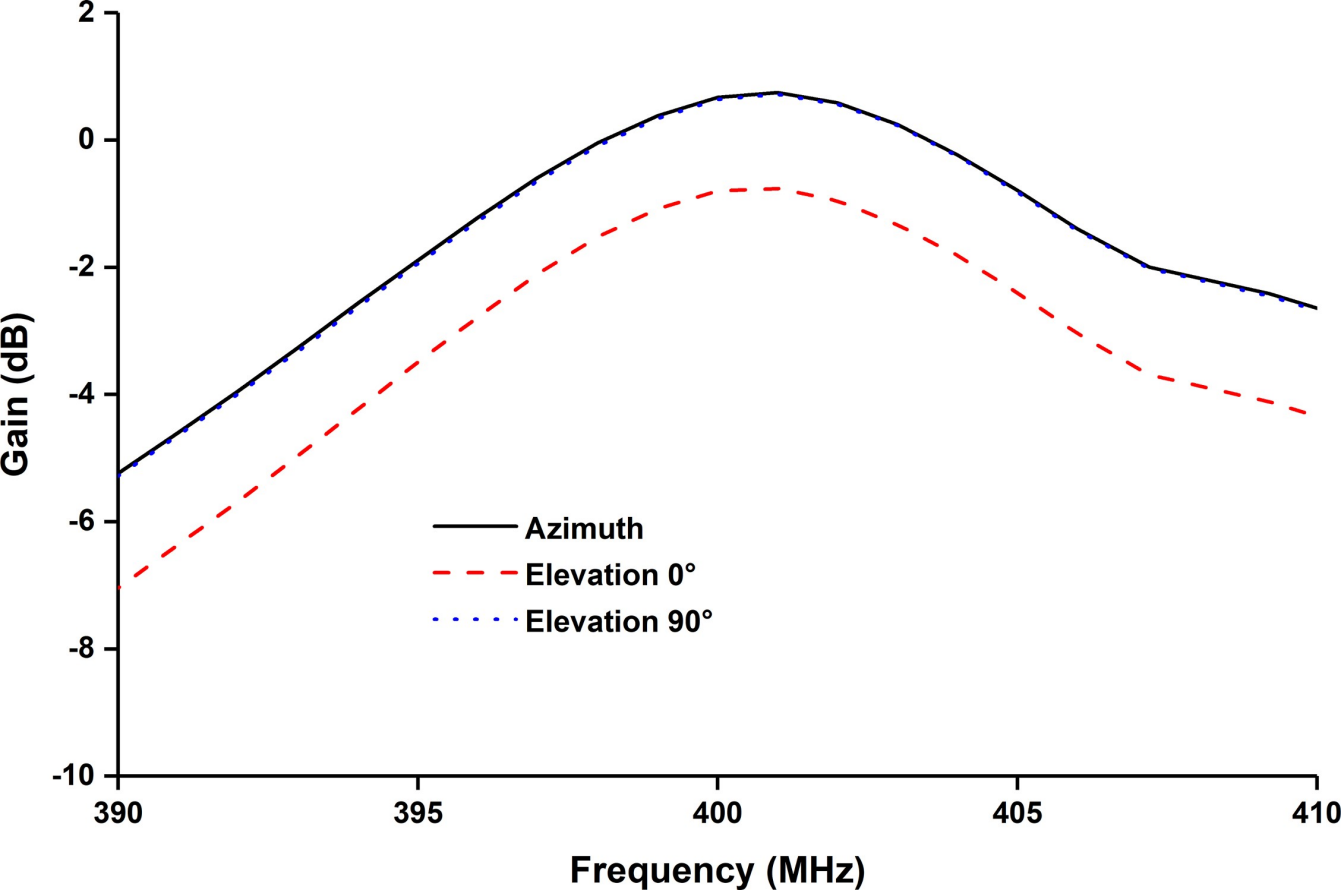
Measured realized gain of the proposed UHF antenna.

**Fig 6:**
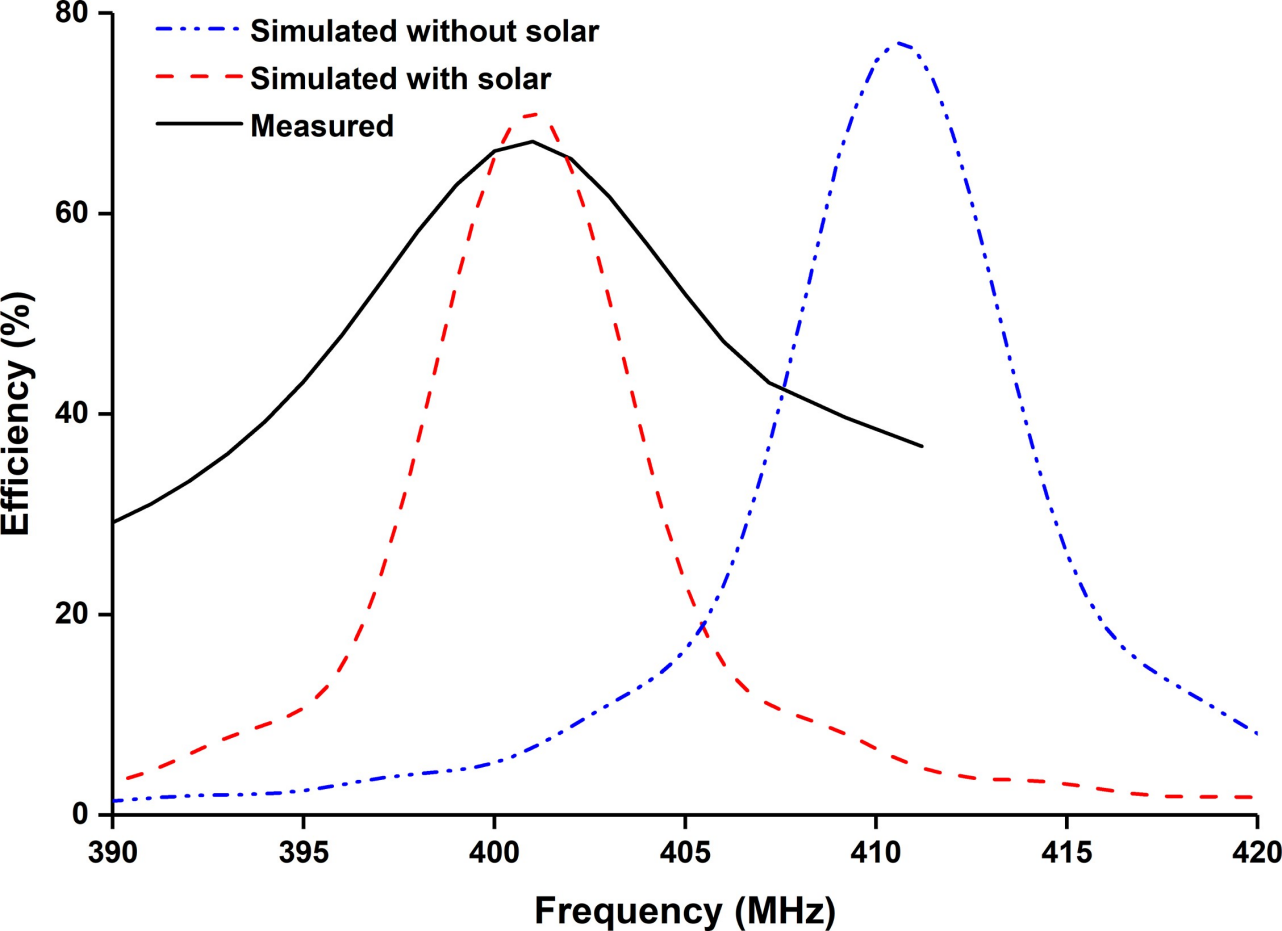
Efficiency of the proposed UHF antenna.

Fig 5 demonstrates the measured and simulated realized gain for both azimuth and elevation planes. The antenna shows maximum realized gain of 1.18dB at 401MHz. The efficiency of the proposed UHF antenna is depicted in Fig 6. Though the lossy nature of solar cells degrades the antenna efficiency, the total efficiency of the proposed UHF antenna at resonant frequency is approximately 62.5% [6].

**Fig 7:**
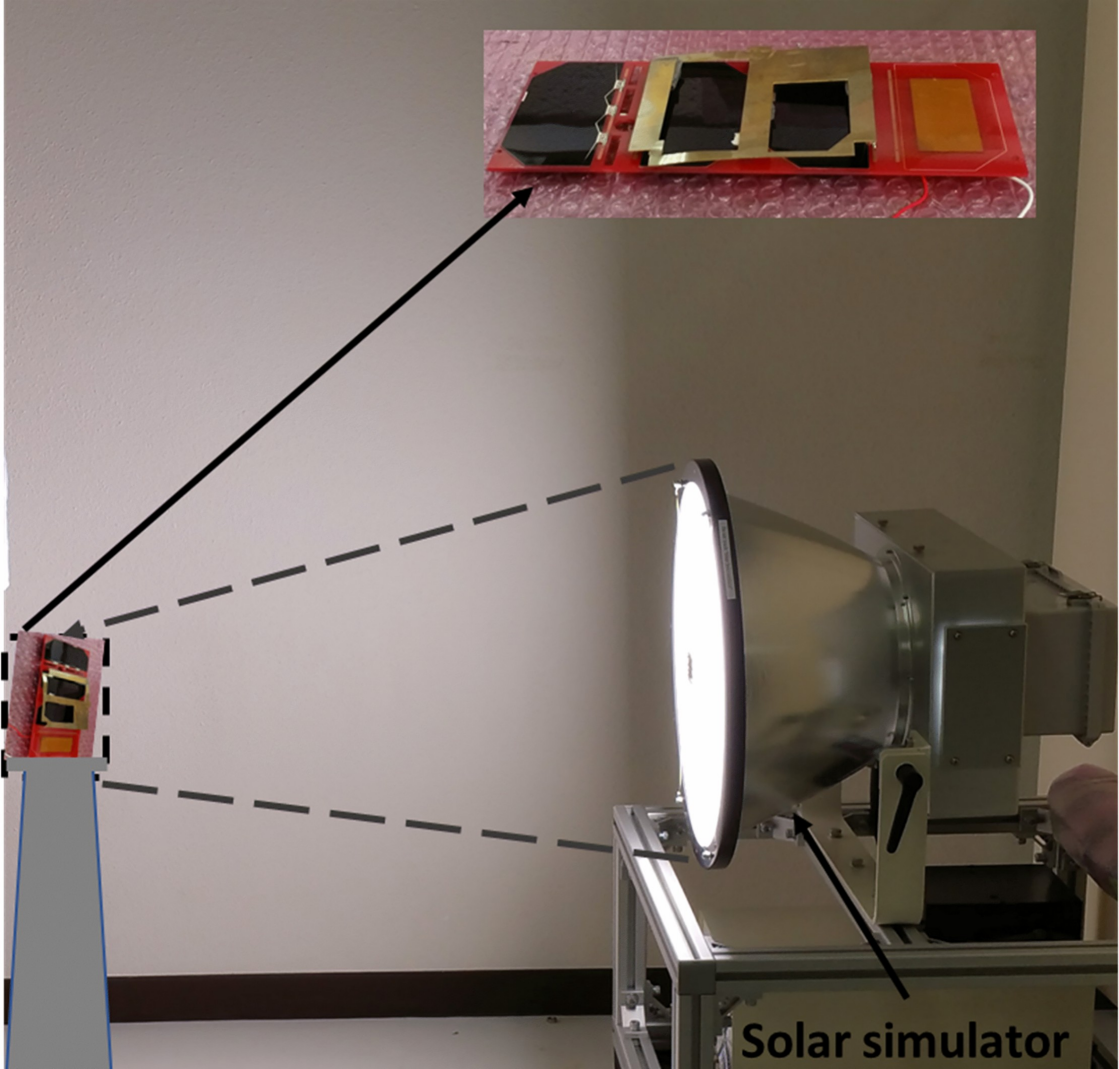
Measurement setup for solar power output investigation with antenna in the Laboratory of Spacecraft Environment Interaction Engineering, KIT, Japan.

Investigation of solar panel output while integrated with the antenna has been performed. The measurement setup is shown in Fig 7. The measurement setup consists of three major components, which are Solar simulator model no. SML-2K1MV1, spectrum analyzer: S-2440C (measuring range 300–1100 nm) and pyranometer: MS-802. The distance between solar simulator and antenna was maintain about 60cm. LEO space quality triple junction GaAs solar cells having approximately 30% efficiency have been used in the measurement, which are connected in the satellite backplane board; and placed below the antenna patch. The output power of the solar panel is measured in free space. After that, the solar panel is placed between the radiating element and the ground plane of the proposed antenna and the output power is measured. The experimental result is presented in Table 2. It is evident that, the antenna integrated solar panel can provide up to 93.95%. of the output power in terms of normal condition in line of sight light penetration. In addition, shadow effects when the satellite rotates with respect to sun has also been investigated by rotating the solar panel 45 degree and 60 degree. At 45^0^ rotation angle, the power output decreased to 0.64 Watt and at 60^0^ rotation angle power output became 0.61 Watt. The percentage of effective power decreased to 69.11% and 65.87% at 45^0^ and 60˚, respectively.

**Table 2:** Experimental data of solar panel output power measurement.

Condition	Solar Output power (W)	% effective power
Open solar panel	0.926	100
Solar panel covered with antenna	0.87	93.95
Antenna structural shadow effect at 45^0^ rotation	0.64	69.11
Antenna structural shadow effect at 60^0^ rotation	0.61	65.87

**Fig 8:**
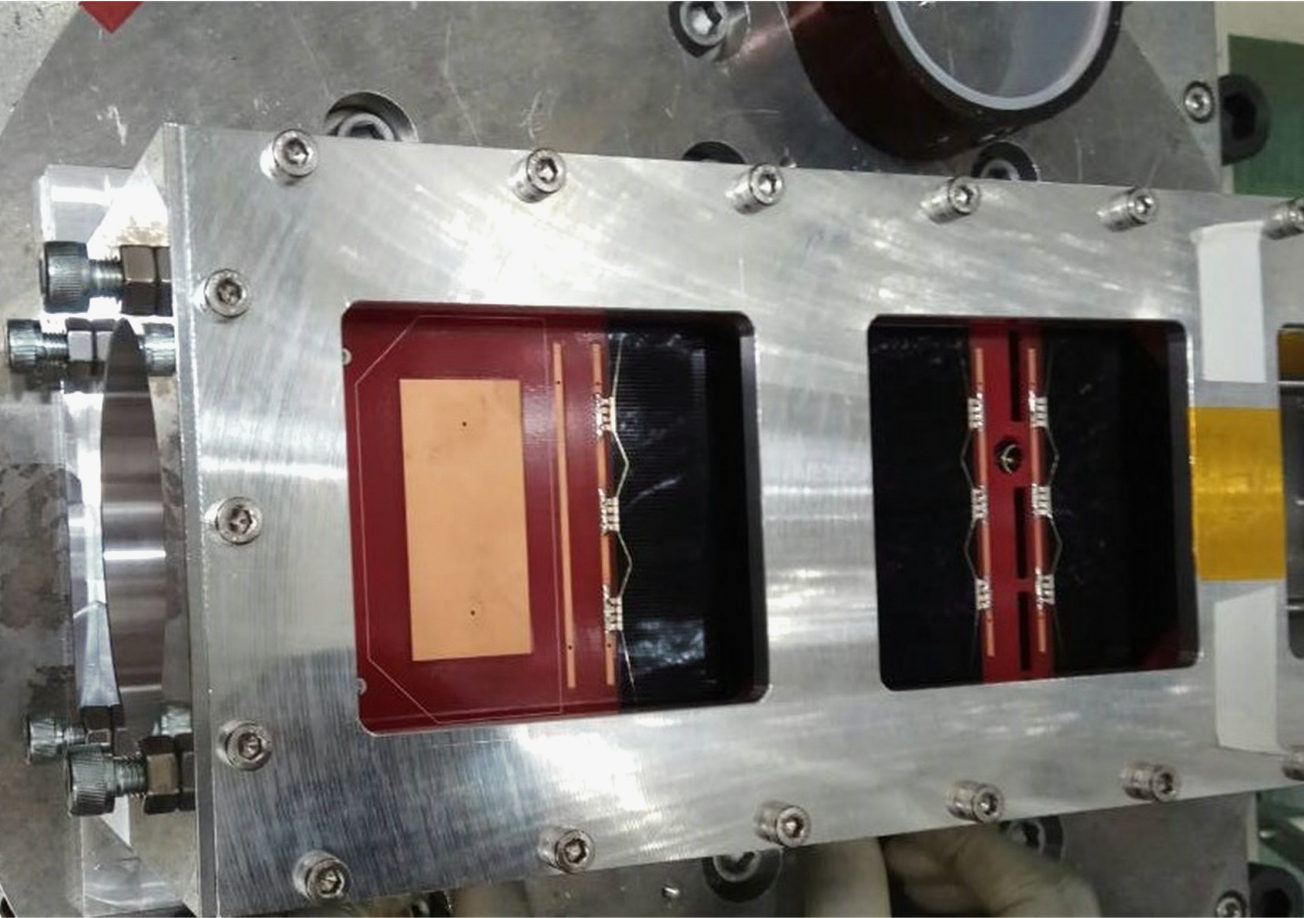
Vibration test setup of 2U satellite body.

**Fig 9:**
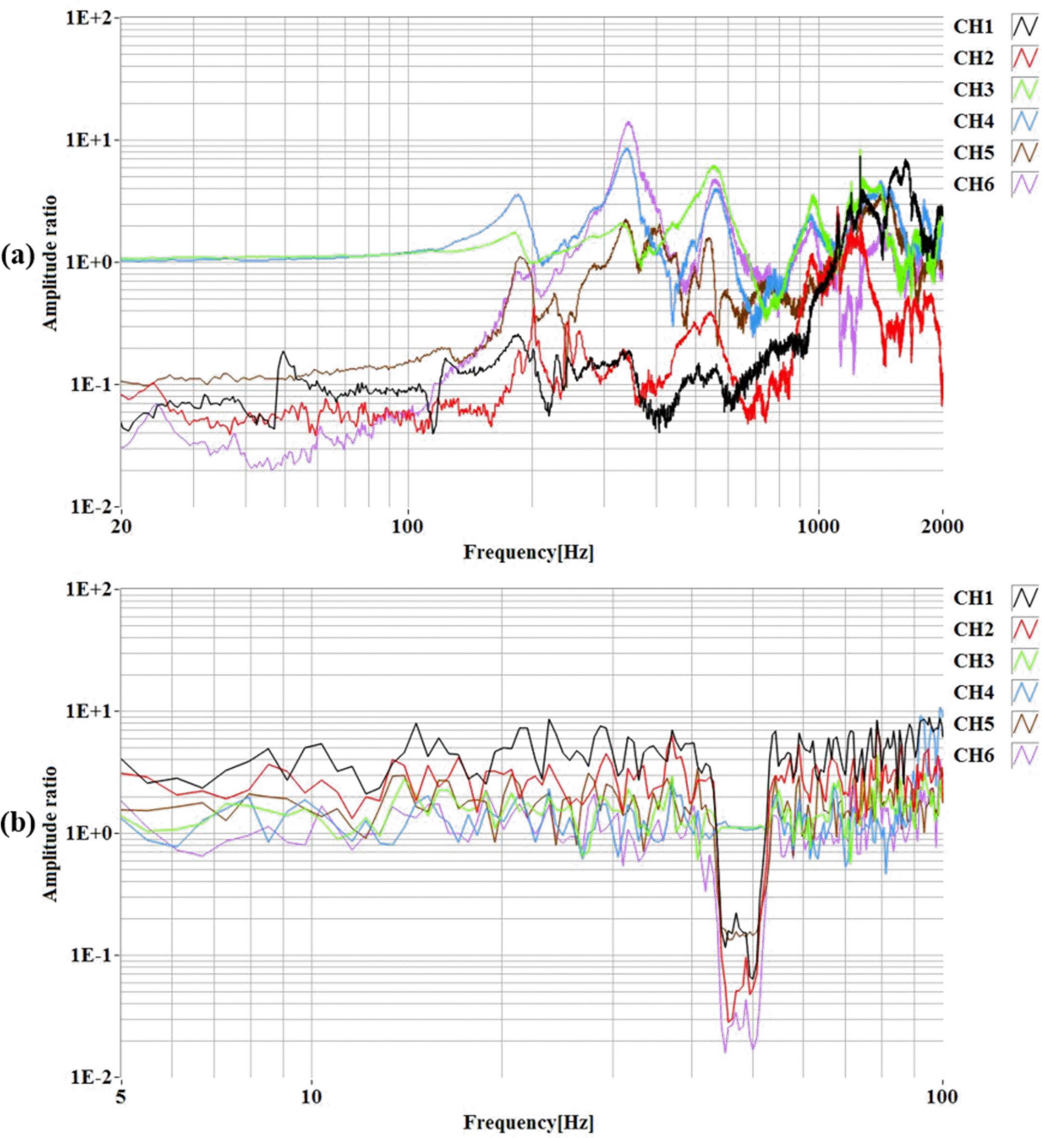
(a). FFT amplitude spectrum before sine burst. (b). FFT amplitude spectrum after sine burst.

To demonstrate the ability of the satellite antenna system to meet prerequisite requirements under vibration conditions which simulate the prediction for flight and survive the launch forces. Vibration test of the nanosatellite structure has been performed while the proposed antenna is integrated with the structure for 20 to 2000 Hz. The same frequency range is often used for low-level sine sweeps. The vibration setup is shown in Fig 8. The first few modes have been observed to identify any structural displacement from Fast Fourier Transform (FFT) spectrum. The FFT amplitude spectrum before and after sine burst has been demonstrated in Fig 9 to verify the vibration test. During excitation and after excitation no malfunction has been observed. Antenna was in exact position as it was before vibration. Moreover, the thermal vacuum tests of the 3D antenna demonstrate the ability of the antenna to meet qualification requirements under vacuum conditions and during high and low temperature which is the expected environment of space. To perform thermal testing, thermocouples are attached to several points inside of the antenna structure. Then the prototype is placed in a test chamber and taken through cycling of temperatures. After performing thermal test, antenna parameters have been measured and compared with existing results. No deviation was observed between two results.

Table 3 illustrates the overall antenna performance. Like the conventional Planar Inverted F Antennas, the proposed antenna facilitates omnidirectional radiation with linear polarization at 401 MHz. The slotted radiating patch provides above 50% open space for light penetration while the solar panels are place in-between the antenna patch and ground plane. It can be seen from Table 4 that; the proposed antenna is a potential candidate for nanosatellite communication system considering the comparison criteria in lower UHF band.

**Table 3:** Antenna performance summary.

Antenna Properties	Value
Operating Frequency	7 MHz (398 MHz– 405 MHz)
Dimension	97 mm× 90 mm × 0.5 mm (Radiating Patch)
Gain	≥ 1 dB
Radiation Efficiency	≥50%
Radiation Pattern	Omni-directional
Open Space for Solar light penetration	≥ 50%
Polarization	Linear

**Table 4:** Comparison with existing UHF antenna.

Reference	Antenna type & Size (mm)	Operating frequency (MHz)	Antenna gain (dB)	Remarks
Liu et al. [11]	Printed patch 320×80×3.17	427.38–437.17	2.12	No solar integration facility Large antenna size and not compatible with 1U and 2U structure
Kakoyiannis et al. [17]	microstrip patch 170×120×6.4	435–437	0.7	No solar integration facility Large antenna size and not compatible with 1U and 2U structure
Podilchak et al. [18]	Microstrip Patch150×150×37	384–410	0.4	Larger antenna size with low gain Large antenna size and not compatible with 1U and 2U structure
Costantine et al. [19]	Deployable helix Helix heigh:500	365	8	High performance antenna but not compatible with 1U structure
ISIS [20]	Monopole 170×3×0.2	UHF	0	Deployable complexity
Proposed antenna	3D-type antenna	398 MHz– 405 MHz	1.18	compatible with 1U and 2U structure solar integration facility Free from deployable complexity

## Conclusion

This paper proposes a nanosatellite body-mounted 3D antenna for UHF communication. The design exclusively features 50% of open space for solar irradiance penetration onto nanosatellite single face where solar panels can be placed. The antenna prototype was fabricated and tested with 1U and 2U nanosatellite structure for performance investigation. The proposed antenna achieved realized gain of 1.18dB and total efficiency of 62.5% with nanosatellite body, which is unique outcomes of the proposed solar integrated UHF antenna.

## Supporting information

S1 File Communication performance test. (DOCX)

S1 Fig Nanosatellite communication testing at 401MHz. (TIF)

S1 Table Attenuation test results. (DOCX)
