# Supplementary material for: Design and compatibility analysis of a solar panel integrated UHF antenna for nanosatellite space mission
Source: PLoS One. 2018 Nov 14;13(11):e0205587. doi: 10.1371/journal.pone.0205587 (PMC6235264; doi:10.1371/journal.pone.0205587)
Supplement: S1 File — (DOCX) [file pone.0205587.s001.docx]

**Communication performance test**

The communication performance of the proposed antenna has been tested with active 2U nanosatellite in anechoic chamber where, equivalent free space path loss has been considered using variable attenuators. The communication test setup is illustrated in following figure. The 2U Satellite was placed on the turn table with COM board transmitting signal through the proposed antenna. The transmitted signal is received by Log periodic antenna and the receiving signal is confirmed by a spectrum analyzer. The signal is received by receiver through a variable attenuator. Attenuation was increased gradually until demodulation was no longer possible. The free space path loss is calculated for both LEO orbital condition and anechoic chamber condition.


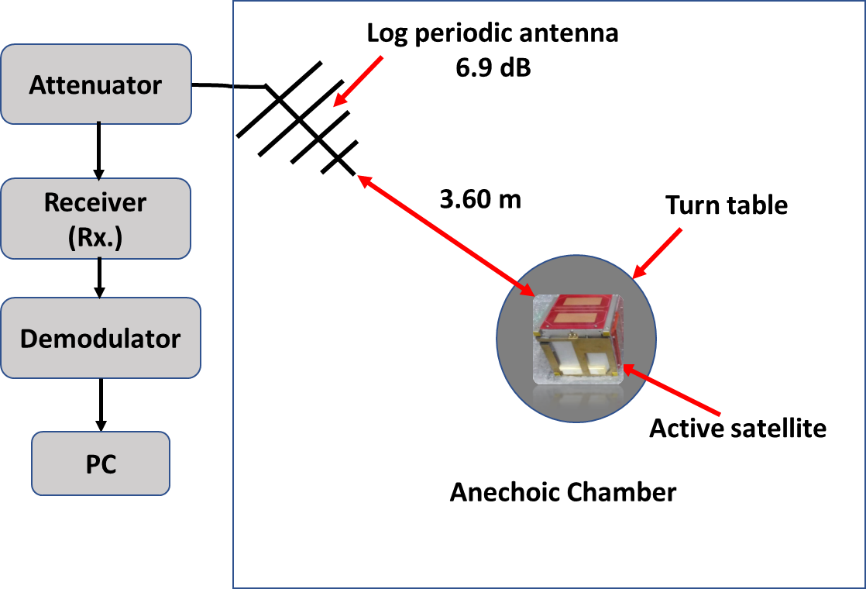


S1 Fig: Nanosatellite communication testing at 401MHz

Free Space Path loss in chamber (FSPL_AC_) at 401 MHz = 27.55 dB

FSPL at orbital altitude (400 km) = 128.5 dB

Gain of the Transmitter Antenna at 401 MHz (G_TX_)=1.18dB

Gain of Log periodic antenna at 401 MHz (G_Rx_) = 6.9 dB

Ground station antenna gain at 401 MHz (G’_Rx_) = 18 dB

Extra attenuation required to achieve signal level= Orbital FSPL – FSPL_AC_ - G’_Rx_ + G_Rx_ = 89.85 dB

S1 Table : Attenuation Test Results

| Nanosatellite Rotation  Angle in Azimuth Plane  (degree) | Maximum attenuation before signal demodulation stopped (dB) |
| --- | --- |
| 0 | 98 |
| 60 | 101 |
| 120 | 105 |
| 180 | 101 |
| 240 | 101 |
| 300 | 94 |
