# Supplementary material for: Design and compatibility analysis of a solar panel integrated UHF antenna for nanosatellite space mission
Source: PLoS One. 2018 Nov 14;13(11):e0205587. doi: 10.1371/journal.pone.0205587 (PMC6235264; doi:10.1371/journal.pone.0205587)
Supplement: S1 Table — (DOCX) [file pone.0205587.s003.docx]

S1 Table : Attenuation Test Results

| Nanosatellite Rotation  Angle in Azimuth Plane  (degree) | Maximum attenuation before signal demodulation stopped (dB) |
| --- | --- |
| 0 | 98 |
| 60 | 101 |
| 120 | 105 |
| 180 | 101 |
| 240 | 101 |
| 300 | 94 |
